# A Challenging Case of Lewy Body Disease in a Symptomatic Patient Negative for Phosphorylated Alpha‐Synuclein: Implications for Lewy Body Spectrum Disorders

**DOI:** 10.1002/alz.092800

**Published:** 2025-01-03

**Authors:** Mohammad Alhneif, Lakshmi Cherukuwada, Mallory Keating, William Cole Corbett, Angelique D. Gonzalez, Oluwaseun Ogunbona, Kevin F. Bieniek, Sudha Seshadri, Shahroo Etemadmoghadam, Margaret E Flanagan

**Affiliations:** ^1^ Glenn Biggs Institute for Alzheimer’s & Neurodegenerative Diseases, University of Texas Health Sciences Center at San Antonio, San Antonio, TX USA; ^2^ Department of Pathology and Laboratory Medicine, University of Texas Health Science Center at San Antonio, San Antonio, TX USA

## Abstract

**Background:**

Lewy bodies (LBs), characterized by intraneuronal inclusions of misfolded alpha‐synuclein (α‐syn) protein, are the pathological hallmark of Parkinson’s disease (PD) and dementia with Lewy bodies (DLB). Because this protein is phosphorylated at serine‐129 in 90% of LBs, its phosphorylation is considered a crucial pathogenic event in LB formation and disease development. Here, we present a unique brain autopsy case of a DLB patient with widespread LBs that were negative for phosphorylated‐α‐syn, challenging traditional diagnostic criteria.

**Method:**

An 86‐year‐old male with a history of agent orange exposure presented with cognitive decline, REM sleep behavior disorder, tremors, and visual hallucinations. Clinical workup was inconclusive, suggesting atypical Parkinsonism or possible DLB. Postmortem neuropathological examination of H&E slides revealed numerous LB‐appearing inclusions in the midbrain. To confirm their identity, immunohistochemical staining for pSYN64, an antibody against phosphorylated‐α‐syn, was performed which showed negative staining (with an appropriately positive control slide which verified that the stain worked). Further immunohistochemistry with antibodies against P62, LB509, and anti‐nitro‐α/β‐synuclein as well as pSYN64 in other brain regions was performed. P62 is regarded as a surrogate marker of α‐Syn inclusions and LB509 is known to stain total α‐syn. Anti‐nitro‐α/β‐synuclein detects nitrated synuclein.

**Result:**

The LB‐like inclusions in all brain regions including the midbrain showed positive staining for P62 and LB509, confirming their α‐synuclein content, but pSYN64 was consistently negative. Anti‐nitro‐α/β‐Synuclein showed scattered staining. The LB disease in this case was neocortical stage, which explains the patient’s cognitive impairment. Additionally, brainstem involvement justifies his parkinsonism during life.

**Conclusion:**

This case challenges the long‐held notion that phosphorylated‐α‐syn is an essential component of LBs especially needed to cause clinical symptoms. The absence of phosphorylated‐α‐syn LBs in a symptomatic patient suggests that additional proteins, or alternative mechanisms of abnormal protein accumulation may be involved in disease pathogenesis. This report highlights the complexity of LB pathology and emphasizes the need for continued research to unravel the diverse protein aggregates and mechanisms underlying LB formation and neurodegeneration. By expanding our understanding of LB spectrum disorders, we can enhance diagnostic procedures and facilitate the development of targeted therapies for patients with these diverse and debilitating conditions.

